# Effects of molecular weight on the optical and electrochemical properties of EDOT-based π-conjugated polymers

**DOI:** 10.1038/s41598-017-01132-5

**Published:** 2017-04-24

**Authors:** Shotaro Hayashi, Shin-ichi Yamamoto, Toshio Koizumi

**Affiliations:** Department of Applied Chemistry, National Defense Academy, 1-10-20, Hashirimizu, Yokosuka, Kanagawa 239-8686 Japan

## Abstract

Absolute molecular weight values obtained by gel permeation chromatography with multi angle light scattering of **PEDOTF8** were approximately 65% of the relative molecular weight values obtained by gel permeation chromatography using polystyrene standards. Both light absorption and molecular weight measurements showed the effective conjugation lengths (absolute molecular weight <2600, relative molecular weight <4000, number of EDOT-F8 units: *n* < ca. 5 unit). The low molecular weight polymers showed higher energy absorption and fluorescence bands. Molecular weight also affected the electrochemical process of the polymer films. The high molecular weight **PEDOTF8** (number average molecular weight >70000) showed a higher redox stability than the low molecular weight one. The two polymers with number average molecular weights of 70200 and 40000 retained 65% and 25% of the charge storage capacity after 100 electrochemical scans, respectively. Square-wave potential step absorptiometry measurements of the polymers with number average molecular weights of 70200 and 40000 showed that the optical contrasts remain stable after ca. 55 and three cycles, respectively. The high molecular weight polymer has a high electrochemical stability and would be a good material for optoelectronic devices.

## Introduction

The optical and electronic properties of π-conjugated polymers are relevant for many applications in the field of semiconductors^1–3^. EDOT, 3,4-ethylenedioxythiophene, is an attractive building unit for producing π-conjugated polymers^4^. The unit enhances the π-conjugation and *p*-doping through specific interactions and its excellent electron-donating property. Thus, PEDOT, poly(3,4-ethylenedioxythiophene-2,5-diyl), and EDOT-based alternating copolymers are high performance organic semiconductors and fluorophores^4^. For this reason, the focus is often placed on the optical (light absorption and fluorescence) and electrochemical (doping–dedoping) in the polymer film. Several parameters of the polymer affect its optoelectronic properties and device performance, such as the degree of polymerisation (molecular weight values)^5–7^ and purity^8, 9^. The molecular weight values of π-conjugated polymers play an important role in the performance of organic field effect transistors and organic solar cells^5–7^. The molecular weight values, including number average molecular weight (*M*_n_) and weight average molecular weight (*M*_w_), and polydispersity of the polymers are of central importance for their applications. Properties such as effective conjugation length and interpolymer coupling within the aggregates as well as the packing behaviour of the polymer chains affect the optoelectronic properties^10–12^. Low molecular weight π-conjugated polymers, which were insufficient effective conjugation lengths, offer higher energy light absorption (band gap) and fluorescence than high molecular weight ones^13, 14^. Thus, low molecular weight fractions of the polydispersive polymers are critically affected to the optoelectronic properties.

Polycondensation is a standard technique for synthesising the π-conjugated polymers. Synthetic pathways of EDOT-based π-conjugated polymers have been developed through palladium-catalysed cross-coupling research^15, 16^. Because the direct arylation reactions have higher atom efficiency and reactivity than the other cross-coupling reactions^17, 18^, the direct arylation polycondensation of EDOT with dibromoarenes have received great interest in recent years^19–22^. High molecular weight EDOT-based π-conjugated polymers (*M*_n_ > 80000) were successfully demonstrated by flow conditions^19^, microwave-assisted conditions^20^ and chloride-promoted conditions^21, 22^. The molecular weight values of poly(EDOT-*alt*-isoindigo) have been reported to affect light absorption properties^19^. Despite the investigation of this donor–acceptor polymer, the relationship between the molecular weight values and the light absorption properties of poly(EDOT-*alt*-fluorene) is still not fully understood. Poly(EDOT-*alt*-fluorene) shows a high FET hole mobility (1.2 × 10^−3^ cm^2^V^−1^s^−1^) and high OPV performance (PCE = 4%) depending on its molecular weight^23^. However, the effects of molecular weight on the fluorescence and electrochemical properties have not been investigated, which presents a challenging topic for understanding polymer properties. In this study, we investigate the light absorption, fluorescence and electrochemical properties of poly(EDOT-*alt*-fluorene) with different molecular weights.

## Results and Discussion

**Figure 1:**
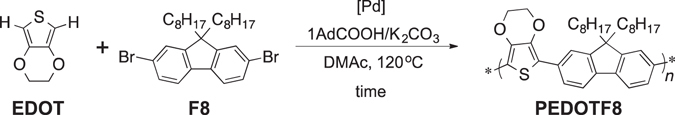
Direct arylation polycondensation of **EDOT** with **F8**.

**Figure 2:**
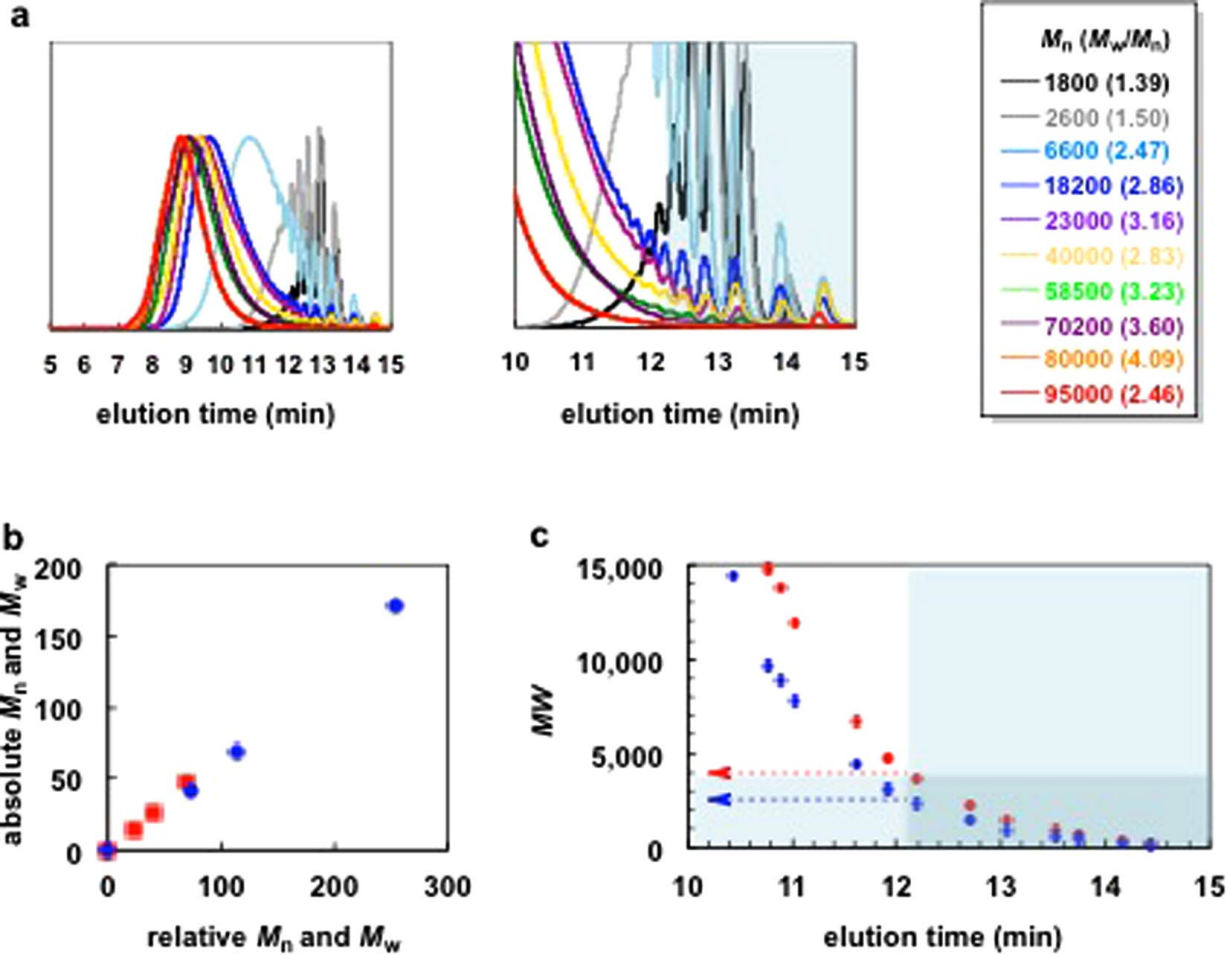
(**a**) Gel permeation chromatography trace of **PEDOTF8** with different molecular weights. (**b**) Plots of relative molecular weight values on absolute molecular weight values. Red: *M*_n_, Blue: *M*_w_. (**c**) Molecular weight values against on gel permeation chromatography elution time. Red: relative *MW*. Blue: absolute *MW*.

Direct (C–H) arylation polycondensation of **EDOT** with 2,7-dibromo-9,9-dioctylfluorene (**F8)** was performed at previously optimised conditions: 1.0 mol% of Pd catalyst (Pd(OAc)_2_, PdCl_2_, PITS-Cl)^21, 22^, 30 mol% of 1-adamantanecarboxylic acid, (1AdCOOH) and 3.0 eq. of potassium carbonate (K_2_CO_3_) in dry *N*,*N*-dimethylacetamide (0.3 M) at 80–120 °C under argon for 5–120 min, giving poly(3,4-ethylenedioxytiophene-2,5-diyl-*alt*-9,9-dioctylfluorene-2,7-diyl), **PEDOTF8**, with different molecular weights (Fig. 1). To systematically evaluate the relationship between properties and molecular weights of **PEDOTF8** different measurements were performed (Fig. 2 and Table 1). The molecular weights of **PEDOTF8** samples were determined by gel permeation chromatography (GPC). The *M*_n_ and *M*_w_/*M*_n_ values of the polymers were 1800–95000 and 1.3–4.1, respectively (Fig. 2a). The measurement using GPC and polystyrene standards showed relative molecular weights (*M*_n_ and *M*_w_), which are not the true (absolute) values because rigid (rod) segments of π-conjugated polymers imitate a large hydrodynamic radius that does not correspond to the molecular weight of polystyrene, which has coil segments. Thus, the average number of repeating unit (*n*) cannot be determined by the relative molecular weight from GPC. Moreover, calculating the *n* value by end-group determination using ^1^H NMR spectroscopy is difficult because the polymer was synthesised via polycondensation. Absolute molecular weight values of the polymers are required for determining *n* value. To estimate the values of absolute molecular weight, GPC with multi angle light scattering^24, 25^ was performed (Table 1). Absolute molecular weight (*M*_n_ and *M*_w_) values of **PEDOTF8** were approximately 65% of the relative molecular weight values (Fig. 2b). This result shows that the rigid π-conjugated polymer segments cause a larger hydrodynamic radius than the coil segments in polystyrene.

**Table 1:** Molecular weights of **PEDOTF8**.

Entry	Relative MW^a^			Absolute MW^b^			Average Number of Unit
	*M*_n_	*M*_w_	*M*_w_/*M*_n_	*M*_n_	*M*_w_	*M*_w_/*M*_n_	*n*_(EDOT-F8)_^*c*^
1	23000	72700	3.16	15000	42200	2.81	28
2	40000	113200	2.83	26000	69900	2.69	49
3	70200	252700	3.60	49000	175900	3.59	93

^a^Estimated by GPC measurements (eluent: THF, standard: polystyrene). ^b^Estimated by GPC-MALS measurements (eluent: THF). ^c^Calculated from *M*_n_ value from absolute measurement.

**Figure 3:**
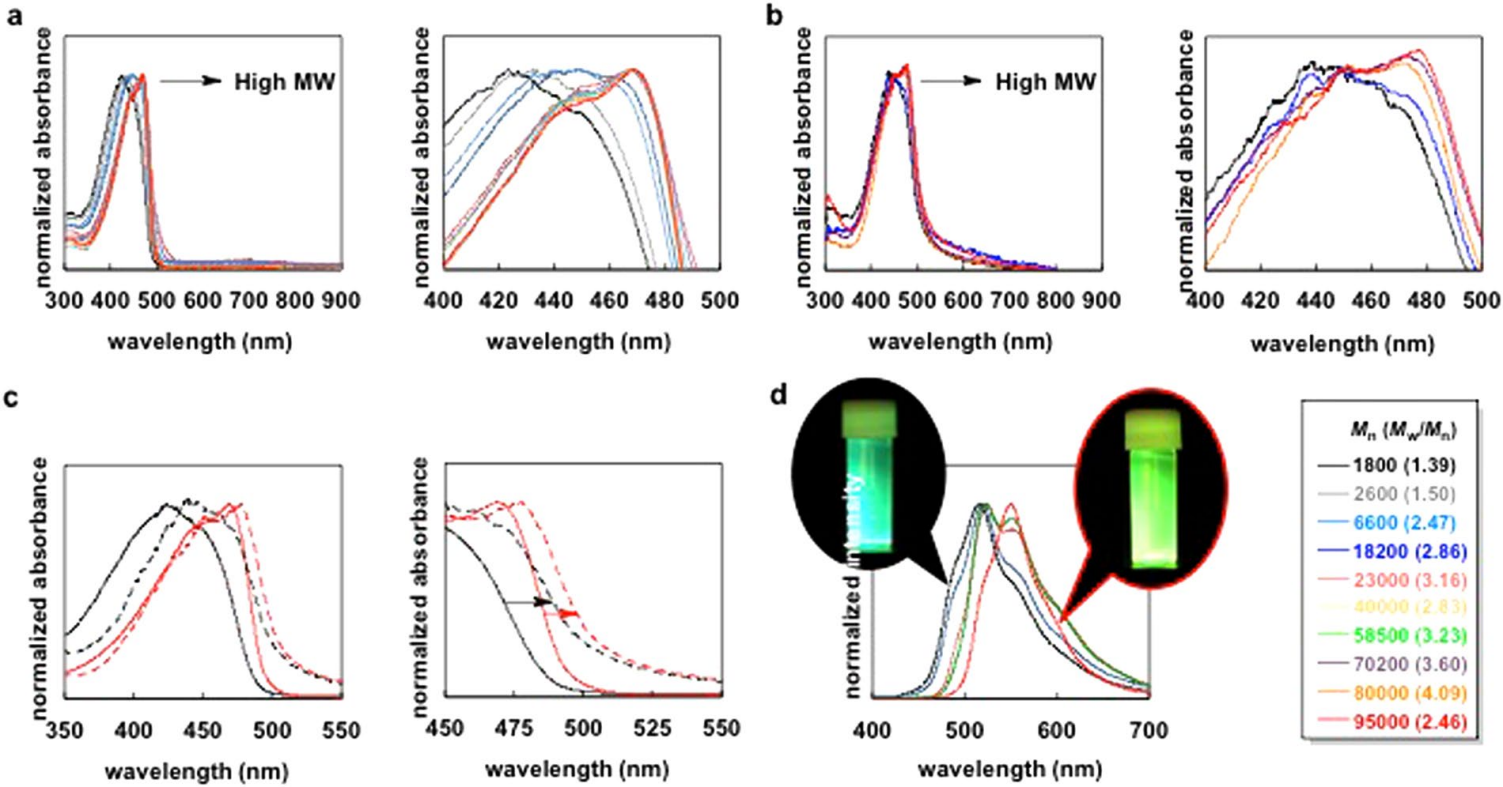
(**a**) UV-vis absorption spectra of **PEDOTF8** with different molecular weights in dichloromethane. (**b**) UV-vis absorption spectra of **PEDOTF8** films with different molecular weights. (**c**) UV-vis absorption spectra of **PEDOTF8** (*M*_n_ = 1800 and *M*_n_ = 95000) in dichloromethane (solid line) and as a film (dotted line). Redshifts are shown by arrows. (**d**) Fluorescence spectra of **PEDOTF8** with different molecular weights in dichloromethane. Photograph of **PEDOTF8** in dichloromethane under UV irradiation. *M*_n_ = 1800 (left) and *M*_n_ = 95000 (right).

Optical properties of the **PEDOTF8** polymers are displayed in Fig. 3. UV-vis absorption bands of **PEDOTF8** in dichloromethane were shifted to a lower energy for increasing *M*_n_ values (Fig. 3a). The band in the film state also showed a bathochromic shift depending on the molecular weight (Fig. 3b). The samples with high *M*_n_ values (70200, 80000 and 95000) showed similar absorption bands in both conditions (Fig. 3a and b), displaying a sufficient effective conjugation length of **PEDOTF8**. The low molecular weight fractions of **PEDOTF8**, which were of insufficient effective conjugation lengths, were not present in these samples. Thus, the low molecular weight **PEDOTF8** samples (*M*_n_ = 1800–58500) include fractions of insufficient effective conjugation lengths (Fig. 2a, right). Because **PEDOTF8** is a low crystallinity (highly amorphous) material, the redshift of absorption band from solution to film is very small^20^. Compared with **PEDOTF8** (*M*_n_ = 95000), **PEDOTF8** (*M*_n_ = 1800) showed a large redshift from solution to film, indicating that the low molecular weight fractions in **PEDOTF8** (*M*_n_ = 1800) form a domain for inter-polymer interaction (Fig. 3c). Fluorescence spectra of **PEDOTF8** in dichloromethane also exhibited different bands depending on the molecular weights (Fig. 3d). The lowest molecular weight sample (*M*_n_ = 1800) showed a low energy fluorescence band peaking at 515 nm (green-coloured fluorescence). However, the high molecular weight samples (70200, 80000 and 95000) showed fluorescence bands peaking at 549 nm (yellow-coloured fluorescence).

Low molecular weight fractions (elution time <12.2 min) were polymers with insufficient effective conjugation lengths (Fig. 2a). The relative molecular weight value of the fraction eluted at 12.2 min was 4000 (Fig. 2c). The absolute molecular weight of this **PEDOTF8** was 2600. The number of EDOT-F8 units (*n*) of the MW was estimated at ca. 5 units. The fractions with an absolute molecular weight below 2600 (relative molecular weight <4000) and less than or equal to five EDOT-F8 units showed dramatically reduced optical properties (Fig. 2a). Therefore, the effective conjugation length of **PEDOTF8** is *n* > 6 units of EDOT-F8.

**Figure 4:**
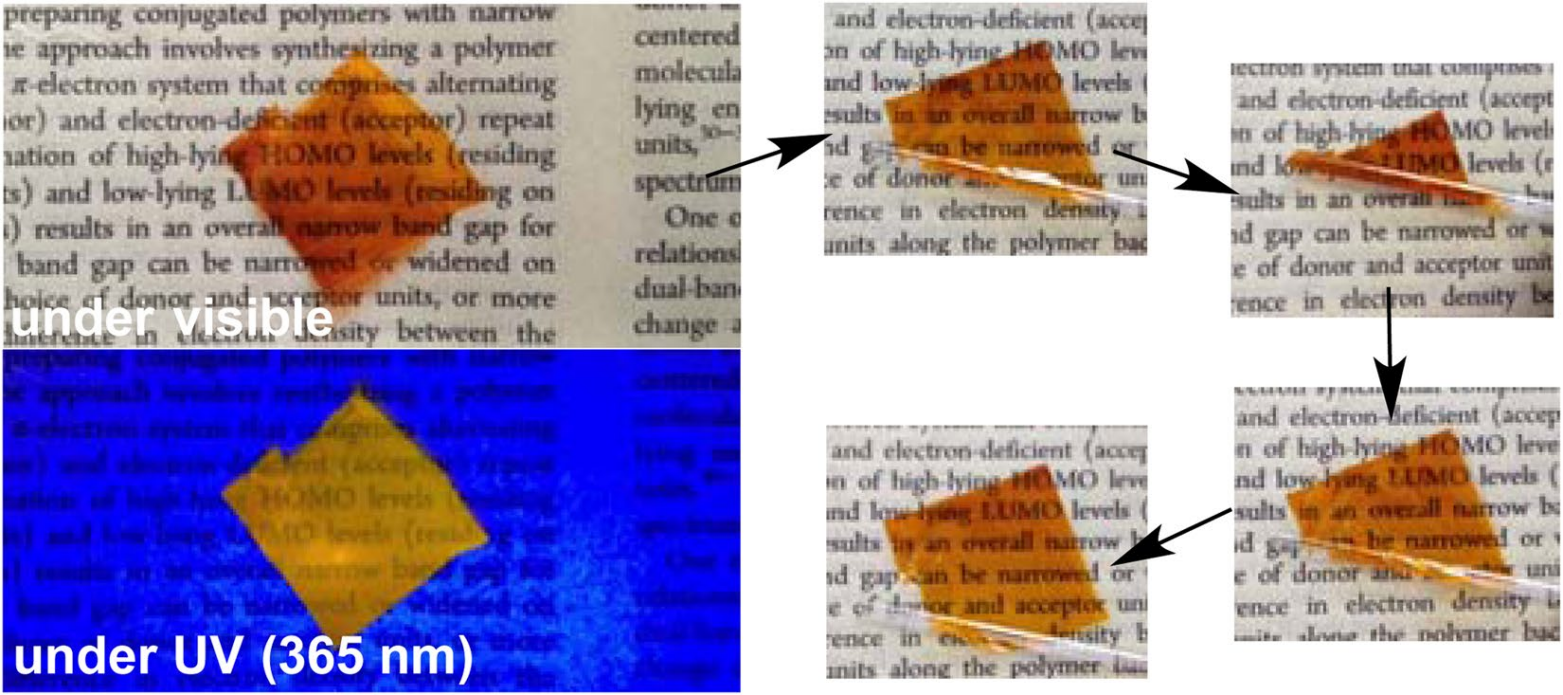
Free-standing **PEDOTF8** film (*M*_n_ = 70200).

The film forming ability and toughness of polymers depend on the length of polymer segment (molecular weight) and crystalinity^26^. Therefore, the film forming ability and toughness of amorphic **PEDOTF8** depend on the molecular weight. Free-standing films of **PEDOTF8** were prepared by casting **PEDOTF8**/toluene solution (10 g/L). The film of high molecular weight **PEDOTF8** (*M*_n_ = 70200) showed tough films suitable for bending and rolling (Fig. 4). However, the molecular weight **PEDOTF8** (*M*_n_ < 40000) produced brittle films.

**Figure 5:**
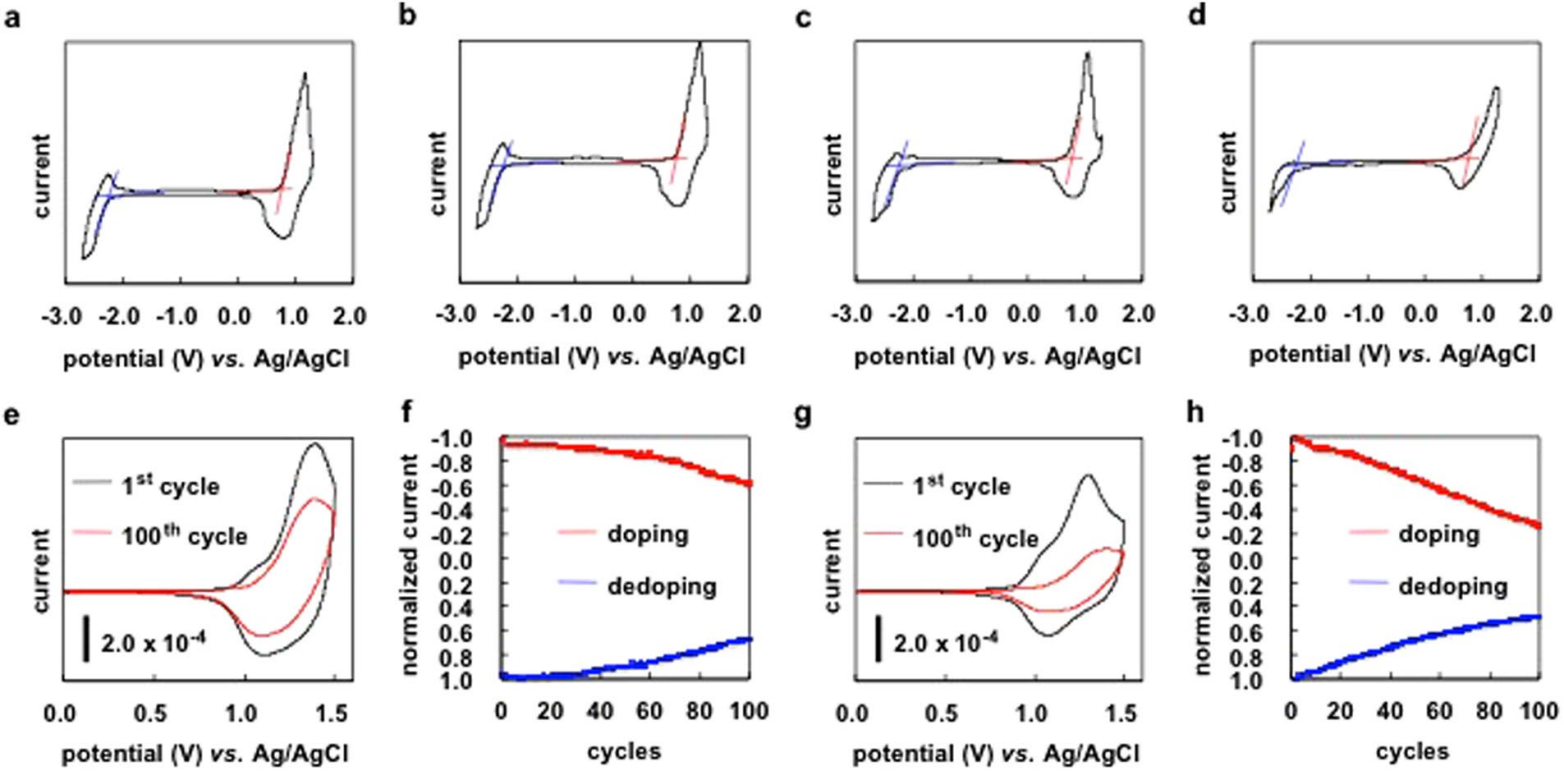
Cyclic voltammograms of **PEDOTF8** on a glassy carbon electrode. (**a**) *M*_n_ = 95000. (**b**) *M*_n_ = 70200. (**c**) *M*_n_ = 40000. (**d**) *M*_n_ = 23000. (**e**) The 1^st^ and 100^th^ cycles of high molecular weight **PEDOTF8** (*M*_n_ = 70200) on the indium-tin-oxide-coated polyethylene terephthalate (ITO/PET) electrode. (**f**) Plots of peak current on cycles of high molecular weight **PEDOTF8** (*M*_n_ = 70200). (**g**) The 1^st^ and 100^th^ cycles of low molecular weight **PEDOTF8** (*M*_n_ = 40000) on the ITO/PET electrode. (**h**) Plots of peak current on cycles of low molecular weight **PEDOTF8** (*M*_n_ = 40000). Conditions: Electrolytic solution of 0.1 M Bu_4_NBF_4_/CH_3_CN, Working electrode: glassy carbon or ITO/PET, Counter electrode: Pt, Reference electrode: Ag/AgCl, Scan rate: 100 mV/s.

Cyclic voltammetry measurements of **PEDOTF8** films on electrode evaluated the doping–dedoping process of counter ions. The process causes a volume change, destroying and detaching the films from the electrode or electrochemical quenching^27, 28^. Reversible current responses of cyclic voltammograms indicate the high electrochemical stability (or toughness) of the films. A high molecular weight polymer shows toughness for volume change because of the entanglement among polymer segments. A three-electrode system equipped with the glassy carbon working electrode, platinum counter electrode and Ag/AgCl as a reference electrode was used in an electrolytic solution of 0.1 M Bu_4_NBF_4_/CH_3_CN. High molecular weight samples (*M*_n_ = 70200 and *M*_n_ = 95000) showed similar *p*- and *n*-doping–dedoping profiles at both oxidation and reduction (Fig. 5a and b). However, the profiles of the lower molecular weight sample (*M*_n_ = 40000) were different and showed irreversible *n*-doping–dedoping (Fig. 5c). Moreover, the cyclic voltammogram of a low molecular weight polymer film showed irreversible response for *n*-doping–dedoping (Fig. 5d). The electrochemical stability of the polymer film was examined by continuous redox cycling^28^. *p*-Doping–dedoping profiles of the 1^st^ and 100^th^ scans, and plots of peak current on scan cycles are displayed in Fig. 5e–h. By comparing the charge storage capacity of the two curves of polymers with *M*_n_ = 70200 and *M*_n_ = 40000, 65% (Fig. 5e) and 25% (Fig. 5g) of the capacities were retained after 100 electrochemical scans, respectively, indicating that the high molecular weight **PEDOTF8** film has a higher electrochemical stability than the low molecular weight **PEDOTF8** and would be a good material for electrochromic devices. Chain ends of polymers often cause decomposition by external stimulation such as chemicals, thermal heating and photo-irradiation^29, 30^. To our knowledge, the electrochemical decomposition of the polymers has not been reported. However, in most of the polymers, inner chains are higher stability than chain ends. The electrochemical stability of **PEDOTF8** probably depends on the chain end (terminal group) of the polymer segment. Low molecular weight **PEDOTF8** in film possesses higher content of the terminal groups than high molecular weight **PEDOTF8**.

**Figure 6:**
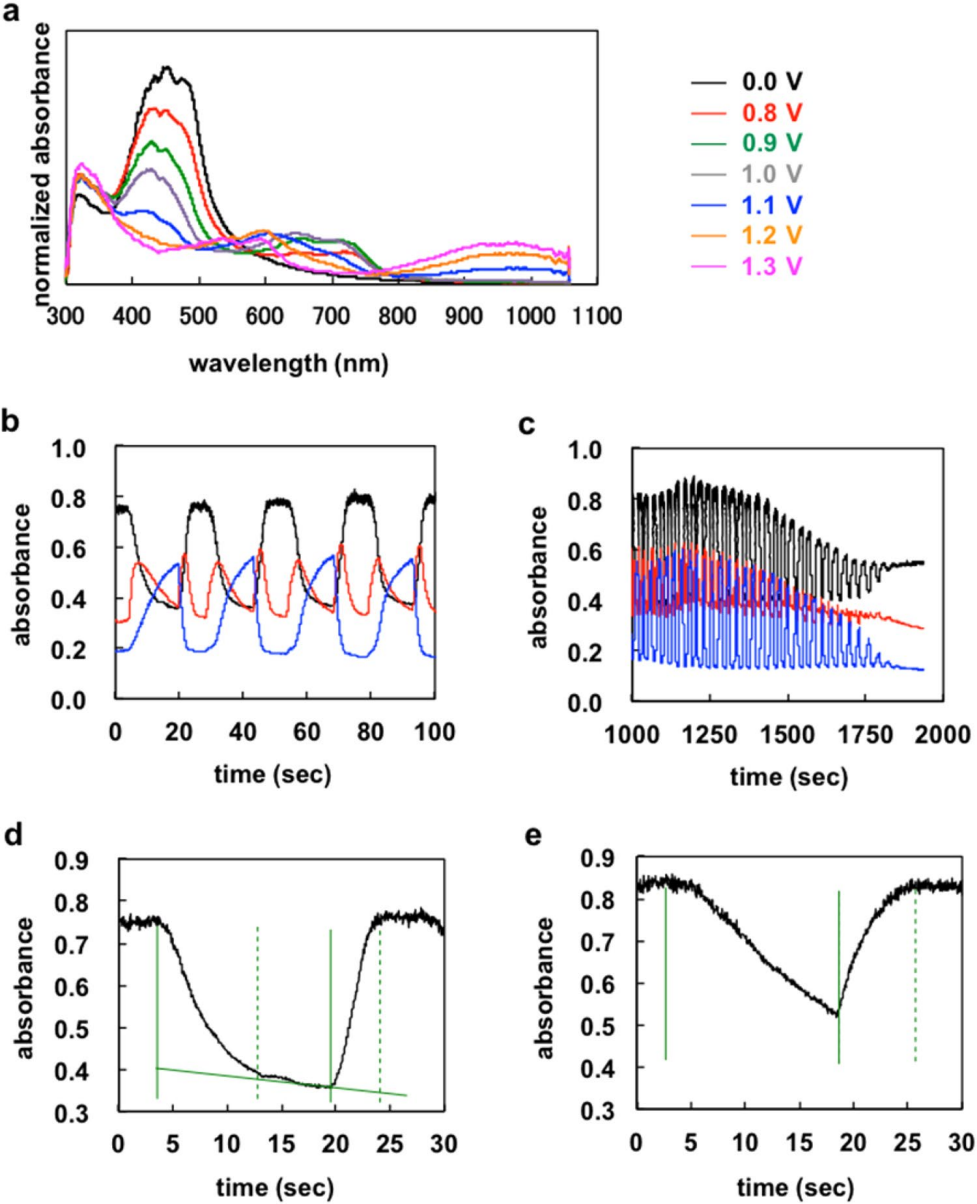
(**a**) Spectroelectrochemistry of **PEDOTF8** (*M*_n_ = 70200) film. (**b,c**) Square-wave potential step absorptiometry of **PEDOTF8** devices in the visible and near infrared regions between 0.0 and 1.3 V with a switching time of 15 s. Black line: 430 nm. Red line: 650 nm. Blue line: 1000 nm. (**d**) Colouring and bleaching processes of the polymer (*M*_n_ = 70200). (**e**) The process of the polymer (*M*_n_ = 40000). Conditions: Electrolytic solution of 0.1 M Bu_4_NBF_4_/CH_3_CN, Working electrode: indium-tin-oxide-coated polyethylene terephthalate, Counter electrode: Pt, Reference electrode: Ag/AgCl.

The spectroelectrochemical changes of **PEDOTF8** were examined by recording the UV-vis absorption spectra at different applied potentials. The drop-cast-coated thin films on an indium-tin-oxide-coated polyethylene terephthalate plate together with the reference Ag/AgCl and counter Pt electrodes were placed in a 1 cm cell. The UV-vis absorption spectra were measured in CH_3_CN containing 0.1 M (*n*-C_4_H_9_)_4_ NBF_4_ at 25 °C. The spectra for the polymer (*M*_n_ = 70200) film are illustrated in Fig. 6a. In the neutral state, the polymer film exhibited only one major π-π* absorption band at 450 nm, producing a yellow colour. Upon progressive oxidation (<1.0 V) of the polymer film, the visible absorption depleted with a concomitant formation of low energy charge carriers, as indicated by the presence of a new band in the lower energy region around 700 nm. This is derived form the generation of polaron^31, 32^. The more oxidation in higher potential generally induces the new bands of bipolaron at different wavelength with decreasing of the band of polaron. Above a potential threshold of >1.0 V, the new bands were observed in the visible and near infrared regions around 600 and 980 nm, respectively, and the intensity at 700 nm decreased. The polymer film revealed a dark green colour in the fully oxidised state, because of the slight tailing of the absorption across the visible region.

The stabilities of the polymer films were evaluated by monitoring the absorbance changes upon repeated redox cycling between the potentials of 1.3 and 0.0 V with a residence time of 30 s. Figure 6b shows the changes during the first four cycles of the devices in both the visible (430 and 650 nm) and near infrared (1000 nm) regions. The **PEDOTF8** (*M*_n_ = 70200) film exhibited long-term stabilities compared with low molecular weight **PEDOTF8** films. The optical contrasts fall steadily at the initial stages and remain stable after ca. 55 repeated cycles (Fig. 6c). The spectral changes in the oxidation process from the neutral state were fully reversible. The switching time was evaluated as 90% of the full switch, because it was difficult to visually perceive any further colour changes beyond this point. The results revealed that the switching times of the colouring and bleaching processes for **PEDOTF8** (*M*_n_ = 70200) film were 8.1 and 4.9 s, respectively (Fig. 6d). The polymer film quickly switched between the neutral states. The electrochromic colour change of **PEDOTF8** (*M*_n_ = 40000) film was slow for the colouring and bleaching processes (Fig. 6e). These results were due to the unstable charge carrier states of the fully oxidised polymer backbone and low toughness of the film.

## Conclusion

In conclusion, we successfully demonstrated the effect of molecular weight on the optical and electrochemical properties of **PEDOTF8**. Absolute molecular weight values obtained by GPC with multi angle light scattering measurements of **PEDOTF8** are approximately 65% of the relative molecular weight values obtained by GPC using polystyrene standards. The incorporation of low molecular weight fractions (absolute *M*_W_ < 2600, relative *M*_W_ < 4000, number of EDOT-F8 unit: *n* < ca. 5 unit) in the polymer affected the absorption and fluorescence properties. The high molecular weight **PEDOTF8** samples showed a higher redox stability of the electrochemical doping–dedoping process of polymer films than the low molecular weight ones, which contributes to the development of a high performance electrochromic device. The molecular weight of the EDOT-based polymers is very important for optoelectronics.

## Experimental

### Materials

3,4-Ethylenedioxythiophene (TCI Japan), 2,7-dibromo-9,9-dioctylfluorene (TCI Japan), 5,5′-dibromo-3,3′-dihexyl-2,2′-bithiophene (TCI Japan), 1-adamantanecarboxylic acid (TCI Japan), K_2_CO_3_ (Kanto), palladium acetate (Wako), palladium chloride (Wako) and dry *N*,*N*-dimethylacetoamide (Wako) were used as received. PITS-Cl was prepared by the reported procedure^22^.

### Synthesis of PEDOTF8 of Different Molecular Weights

A mixture of EDOT (43 mg, 0.30 mmol), 2,7-dibromo-9,9-dioctylfluorene (164 mg, 0.30 mmol), 1-adamantanecarboxylic acid (16 mg, 30 mol%), K_2_CO_3_ (125 mg, 0.90 mmol) and 1.0 mol% of palladium (Pd(OAc)_2_, PdCl_2_, PITS-OAc, PITS-Cl) was stirred in dry *N*,*N*-dimethylacetoamide (1.0 mL) for 5–120 min at 80–120 °C under argon. The reaction mixture was diluted by toluene, rapidly cooled to room temperature, and then filtered to remove insoluble salts. The filtrate was poured into a large amount of methanol. The resulting polymer was collected by filtration and washed with a large amount of methanol. The polymer was dried under vacuum, producing an orange powder. The measurements of the polymer were performed without further treatment. The yield was estimated by the weight of the polymer, which was insoluble in methanol.

#### PEDOTF8

Yellow-coloured powder. ^1^H NMR (300 MHz, CDCl_3_): *δ* 7.9–7.6 (*Ar-H*, br), 6.31 (end group of EDOT unit), 4.45 (O(C*H*_*2*_)_2_O, br), 2.05 (C*H*_*2*_(CH_2_)_6_CH_3_, br), 1.25–0.77 (CH_2_(C*H*_*2*_)_6_C*H*_*3*_, br). ^13^C NMR (75.45 MHz, CDCl_3_): *δ* 151.4, 139.5, 138.6, 131.7, 125.0, 120.3, 119.7, 116.1 64.6, 55.2, 40.4, 31.8, 30.0, 29.2_4_, 29.2_1_, 23.8, 22.6, 14.1. Anal. Calcd. for (C_35_H_44_O_2_S)_n_: C, 79.50; H, 8.39. Found: C, 79.28; H, 8.32.

### Measurements

Liquid-state ^1^H and ^13^C NMR spectra were recorded on a JEOL EX-300 spectrometer. Elemental analyses were performed on a Thermo Finnigan Flash EA1112 CHN-O analyser. GPC analyses were performed by a Toso GPC system (HLC-8220), using tetrahydrofuran as the eluent after calibration with polystyrene standards. GPC–MALS measurements were taken in THF at 25 °C (column temperature: 40 °C) using a Dawn EOS instrument (Ga-As laser, 1⁄4690 nm) to evaluate absolute *M*_n_ and *M*_w_ of the polymers. The specific refractive index increment (q_n_/q_c_), which is necessary for the analysis of GPC with multi angle light scattering, was measured with an Otsuka Electric DRM-3000 (1⁄4633 nm) at 25 °C. The measured q_n_/q_c_ of **PEDOTF8** in THF was 0.5935 cm^3^ g^−1^. UV-vis absorption spectra were obtained on an Ocean Optics USB4000-XR1 fibre spectrometer with DH2000-BAL tungsten halogen light source. Fluorescence spectra were obtained on an Ocean Optics USB4000 fibre spectrometer with PX-2 pulsed xenon light source. Cyclic voltammetry measurements were performed by ALS 611. A three-electrode system equipped with the glassy carbon or indium-tin-oxide-coated polyethylene terephthalate electrode, platinum counter electrode and Ag/AgCl as a reference electrode was used in an electrolytic solution of acetonitrile containing 0.1 M tetraethylammonium tetrafluoroborate.
